# The Psychology of Uncertainty and Three-Valued Truth Tables

**DOI:** 10.3389/fpsyg.2018.01479

**Published:** 2018-09-04

**Authors:** Jean Baratgin, Guy Politzer, David E. Over, Tatsuji Takahashi

**Affiliations:** ^1^CHArt (P-A-R-I-S), Université Paris 8 & EPHE, Paris, France; ^2^Institut Jean Nicod, École Normale Supérieure, Paris, France; ^3^Psychology Department, Durham University, Durham, United Kingdom; ^4^School of Science and Engineering, Tokyo Denki University, Tokyo, Japan

**Keywords:** natural language connectives, three-valued truth tables, uncertainty, de Finetti's tri-event, subjective probability

## Abstract

Psychological research on people's understanding of natural language connectives has traditionally used truth table tasks, in which participants evaluate the truth or falsity of a compound sentence given the truth or falsity of its components in the framework of propositional logic. One perplexing result concerned the indicative conditional *if A then C* which was often evaluated as true when *A* and *C* are true, false when *A* is true and *C* is false but irrelevant“ (devoid of value) when *A* is false (whatever the value of *C*). This was called the “psychological defective table of the conditional.” Here we show that far from being anomalous the “defective” table pattern reveals a coherent semantics for the basic connectives of natural language in a trivalent framework. This was done by establishing participants' truth tables for negation, conjunction, disjunction, conditional, and biconditional, when they were presented with statements that could be certainly true, certainly false, or neither. We review systems of three-valued tables from logic, linguistics, foundations of quantum mechanics, philosophical logic, and artificial intelligence, to see whether one of these systems adequately describes people's interpretations of natural language connectives. We find that de Finetti's (1936/1995) three-valued system is the best approximation to participants' truth tables.

## Introduction: the bayesian approach to the psychology of reasoning

From the beginning of their investigations, and for nearly a century, psychologists studying human deductive reasoning considered bi-valued logic as the sole frame of reference. Their early inspiration was limited to Aristotelian syllogistic (Binet, 1902; James, 1908) but in the 1950s Piaget adopted propositional logic which he assumed to be the basis of adults' cognitive functioning (Inhelder and Piaget, 1958). The elementary connectives of natural language for negation, conjunction and disjunction were identified with the logical connectives ¬, ∧, and ∨, respectively, and the indicative conditional *if A* (antecedent)*, then C* (consequent) was identified with the material conditional (or implication *A*⊃ C). However, in 1966, Wason observed that when people are required to make judgments about conditionals in terms of true and false, they often produce a table that differs from the material conditional. Participants consider that a conditional *if A then C* is made “true” by the *A and C* state of affairs and made “false” by the *A and not-C* state, but that the *not-A* cases (*not-A and C* and *not-A and not-C*) are “irrelevant” to the truth value of *if A then C*. Psychologists came to call this truth table “defective” to underscore participants' apparent imperfect comprehension of the material conditional which was assumed to be the meaning of *if … then*. This “defective” conditional is represented in Table 1 (column 1) as C|_″*d*″_*A*. Wason's (1966) observation was confirmed by early experimental studies in which part of the participants required to choose or construct the states of affairs that make the sentence true or false disregard the *not-A* states (Evans, 1972) or choose the option *irrelevant* when it is offered to them (Johnson-Laird and Tagart, 1969), or spontaneously express the irrelevance of these cases (Delval and Riviére, 1975; Politzer, 1981). Whatever this table is called, it should be contrasted with the truth table for the material conditional which is true in the *not-A* cases (see Table 1, column 2). In addition, a “defective” biconditional (denoted by C‖_″*d*″_*A* in Table 1, column 3) has also been observed (Delval and Riviére, 1975). It is made true by the *A and C* state of affairs, and made false by the *A and not-C* and *not-A and C* states, with the *not-A and not-C* state alone “irrelevant” (see Evans and Over, 2004, for further research on the “defective” conditional and biconditional truth tables in psychology).

**Table 1:** The different truth tables for the conditional if A then C: two-valued (columns 1–3′) and three-valued (columns 4–7).

		**1**	**1′**	**2**	**3**	**3′**			**4**	**5**	**6**	**7**
**A**	***C***	**C|_″*d*″_A**	***C*|_*Fi*_*A***	***A*⊃*C***	**C‖_″″*d*_*A***	***C*‖_*Fi*_*A***	**A**	***C***	***C*|_?_*A***	***C*|_*Fi*_*A***	***C*|_*Fa*_*A***	***C*|_*C*_*A***
T	T	T	T	T	T	T	T	T	T	T	T	T
							T	∅	?	∅	∅	∅
T	F	F	F	F	F	F	T	F	F	F	F	F
							∅	T	?	∅	∅	T
							∅	∅	?	∅	∅	∅
							∅	F	?	∅	F	F
F	T	I	∅	T	F	F	F	T	∅	∅	∅	∅
							F	∅	?	∅	∅	∅
F	F	I	∅	T	I	∅	F	F	∅	∅	∅	∅

T, true; F, false; I, Irrelevant; ∅, third value; ?, T or F or ∅;

1. (C|_″*d*″_*A*), the 2 × 2 “defective” conditional table;

1′. (C|_Fi_A), the 2 × 2 Finettian interpretation of 1;

2.(A⊃C), the 2 × 2 material conditional;

3. (C‖_″*d*″_*A*), the 2 × 2 “defective” biconditional table;

3′. (C||_Fi_A), the 2 × 2 Finettian interpretation of 3;

4. (C|_?_A), the 3 × 3 general (underspecified) conditional;

5. (C|_Fi_), the 3 × 3 de Finetti conditional table;

6. (C|_Fa_A), the 3 × 3 Farrell conditional table;

*7. (C|_C_A), the 3 × 3 Cooper conditional table*.

Until the end of the century, the major part of the theoretical debate on deduction in cognitive psychology revolved around the format of representation of the connectives. For one stream of research the representation was assumed to be syntactic and deduction rule-governed (Rips, 1994; Braine and O'Brien, 1998) whereas for another stream it was assumed to be semantic and deduction model-based (Johnson-Laird and Byrne, 1991). Whatever the option may be, the frame of reference was still two-valued logic and the explanation of the defective table was a major item on the agenda. However, in recent years, this old model of reference has been questioned and a new approach using a probabilistic frame of reference has emerged. This new paradigm in the psychology of reasoning emphasizes that most human inferences take place when there is some degree of uncertainty about the subject matter (Oaksford and Chater, 2007, 2009; Over, 2009, 2016; Pfeifer and Kleiter, 2010; Evans, 2012; Elqayam and Over, 2013; Pfeifer, 2013; Baratgin et al., 2015; Baratgin and Politzer, 2016; Over and Baratgin, 2017; Over and Cruz, 2018). This uncertainty is found in both everyday thought and scientific inference, when people are trying to decide what they will most enjoy on a lunch menu, or to infer what has caused an outbreak of food poisoning.

This new Bayesian approach to the psychology of reasoning has received great impetus from two sets of experimental findings. The first finding is that, as claimed by the theory, people generally judge the probability of the indicative conditional, *P(if A then C)*, to be the conditional probability of *C* given *A, P(C|A)* (for early data see: Evans et al., 2003, 2007; Oberauer and Wilhelm, 2003, but see also Douven and Verbrugge, 2010; Vidal and Baratgin, 2017). The second finding is that participants' assessments of the conclusions of explicit deductive inferences made under uncertainty tend to be in the *coherence intervals* determined by the probability of the premises, that is, participants tend to conform to the laws of probability (Pfeifer and Kleiter, 2009, 2010, 2011; Pfeifer, 2014; Singmann et al., 2014; Cruz et al., 2015; Evans et al., 2015; Politzer and Baratgin, 2016).

An important target of the new Bayesian paradigm concerns the “defective” table mentioned earlier. Supporters of the new paradigm consider that far from being anomalous it reveals a semantics that differs from the material conditional (Baratgin et al., 2013, 2014)^1^. This point will be developed below and generalized to the basic connectives of natural language (negation, conjunction, disjunction) and also to the biconditional.

## The de finettian approach

Is there a normative framework for unifying all these experimental results? We have argued (Baratgin, 2015; Baratgin and Politzer, 2016; Over and Baratgin, 2017; Over and Cruz, 2018) that de Finetti's Bayesian subjective theory offers just such a framework. De Finetti is one of the founding fathers of modern probability theory, and the most prominent representative of subjective Bayesianism. His overall approach to probability (de Finetti, 1974) has deep psychological relevance (see Baratgin and Politzer, 2006, 2007; Baratgin, 2015, for a discussion in the field of the psychology of probability judgment). His conception of probability as subjective degree of belief, and of the assessment of probability through the well-known betting procedure, are rooted in psychological reflection.

de Finetti (1980) proposed three levels of knowledge of an event. The *objective* level*, Level 0*, corresponds to binary logic, in which every statement that expresses the occurrence or the non-occurrence of an event is objectively true or false. This is the level of events that are known for sure. It is this level that was traditionally studied in the psychology literature of reasoning, even though it is severely limited for a psychological approach, for people often do not know for sure what is true and what is false. It is also, ironically, the level of which de Finetti (2006, p. 113) says that it is “sterile” because logic has no other use than order, enumerate, and expound what is already known. A purely logical science cannot be concerned in forecasting. Hence the need to substitute this “rigid logic” with a ”logic of the probable“ that is the logic of everyday allowing to make predictions with regard to uncertain knowledge (de Finetti, 1977/1993, p. 494).

Beyond Level 0, de Finetti (1980) considered two other levels that are *subjective*. On *Level 1*, the event (or statement) concerns a specific object defined by its own characteristics known to the individual. An event is always conditioned on the individual's personal state of knowledge. The statements can be classified as having one of three values: *true*, characterizing an expected event that has happened; *false*, characterizing an expected event that has not happened; and *uncertain*. The value *uncertain* is to be understood as follows. It represents the subjective point of view of an individual who is wondering whether or not an event will happen or, equivalently, whether the statement that expresses the occurrence of the event is true or false. The third value reflects a transitory state of *ignorance* (at a given time) until the statement is verified or falsified. Until this takes place, it is impossible to give it a truth value. Even though he did not vary in this conception of the third value, he used various terms to designate it; his favorite expression was “void” (e. g., de Finetti, 1967, 1974, 1995/2008) which we will adopt and will denote by “∅”.

To formalize these notions, de Finetti (1936/1995, 1967, 1974, 1995/2008, 2006) defined a three-valued system that uses the third value *void* and is *superimposed* on a two-valued logic that uses *true* and *false*. We describe below the three-valued truth tables that define this system, specifying how these values are propagated for the usual connectives.

The second epistemic level in de Finetti (1980), *Level 2*, is a development of the first level. At this level, the initially non-numerical degrees of belief are finally expressed as numerical probability judgments. People are seldom fully ignorant about an event. They have expectations, make subjective probability judgments, engage in wagers, etc. This level corresponds to the full range of subjective degrees of belief about events where the initial ignorance and the ensuing uncertainty give way to the expression of additive probabilities. Fine distinctions are thus possible at Level 2, which is of psychological importance, since both ordinary people and scientists do often distinguish between events that are uncertain, judging some as more probable than others conditionally on their personal state of knowledge (Baratgin, 2015).

A substantial amount of research on uncertain reasoning has been carried out at Level 2—in fact most of the work mentioned above on the probability of conditionals or deduction under uncertainty. Hardly any research has been done to investigate Level 1 (with the exception of Baratgin et al., 2013, considered below). Level 1 should support and lead up to Level 2, and yet most contemporary theorists in the de Finetti tradition have concerned themselves with a much more refined and expressive system at Level 2 in which the third value for *if A then C* is specified by the conditional probability itself, *P(C|A)*, and the logical values *true* and *false* are replaced with 1 and 0 (Gilio, 1990; Jeffrey, 1991; Coletti and Scozzafava, 2002; Pfeifer and Kleiter, 2009). As Baratgin et al. (2013) point out, Level 2 removes some anomalies in Level 1, for people are never ignorant of trivial tautologies, such as *if A & C then A* and *A or not-A* (Over and Baratgin, 2017). But people do not always, and could not always, make such fine-grained evaluations of Level 2. They can, however, simply express their ignorance, or in other words, can remain at the transitory level 1. In summary, there is a gap to fill. De Finetti's theory has gained much experimental support at Level 2, but the question of its descriptive adequacy at Level 1 is open. The present paper addresses this question in several experiments, our aim being to test the descriptive adequacy, for ordinary people's judgments, of de Finetti's Level 1 in his overall theory of subjective probability. For half a century research in the psychology of reasoning has produced robust results on the comprehension of the connectives of propositional logic. People's performance indicates that they possess negation and conjunction, and to a lesser extent, disjunction (Manktelow, 2012) but their comprehension of the material conditional and biconditional is “defective,” in the sense mentioned above. However, these studies were limited to the framework of classical bi-valued logic. The change of conceptual framework brought about by the Finettian theory necessitates that these studies be carried out with a tri-valued logic. The present study applies itself to refine and reinterpret the old results.

We now turn to the analysis of de Finetti's three-valued system in some detail. We begin with focusing on the conditional, which leads us to the concept of *conditional event* (or tri-event). A conditional event is defined by de Finetti (1936/1995) as a logical entity that is true when the antecedent A and the consequent C are true; false when A is true and C false; and void in the sense introduced above when A is false. The conditional event is closely analogous to a conditional bet, which is won in the first case, lost in the second case, and called off in the third case, when it is “void” and no one wins or loses (see Politzer et al., 2010, on this analogy and the relation to Ramsey, 1926/1990, 1929/1990). So, at Level 1, the betting interpretation helps illustrate the void case^2^.

It now appears that the empirical “defective” table for the conditional should be called the “2 × 2 de Finetti table” (and similarly for the “defective” biconditional) to avoid the negative term “defective” (Milne, 2012; Baratgin et al., 2013, 2014; Nakamura and Kawaguchi, 2016). Notice (Table 1) that in columns 1 and 3 the empirical “defective” table bears a symbol ”I“ (for *irrelevant*), whereas in column 1′ and 3′ the 2 × 2 de Finetti tables bear the symbol “∅” (for *void*). This point deserves explication. The 2 × 2 “defective” table in column 1 (Table 1) describes the psychological observation that participants judge that the states of affairs in which the antecedent is false do not allow to evaluate the conditional sentence in terms of true or false. Participants say that the sentence is neither true nor false, or that it may be true or false, or that one cannot know, and the term “irrelevant” (readily endorsed by participants) was coined by psychologists to express participants' perplexity about the truth value of the sentence. In other words, “irrelevant” and “void” refer to the same state of ignorance, the former being empirically-based and descriptive, and the latter theoretical.

The next step is to take into account the ignorance that can affect elementary events, considering that they, too, can be true, false or void (because for de Finetti all events are conditional), leading to a three-valued (3 × 3) truth table for the conditional event (denoted by *C*|_*Fi*_*A* in Table 1, column 5) which de Finetti (1936/1995) called ”subordination“. Similarly, he defined three-valued truth tables for the ordinary connectives (negation, conjunction, disjunction, see below). This set of truth tables which we will call “3 × 3 de Finetti tables” constitutes *de Finetti's Level 1 system*, abbreviated to Fi.

Traditional psychological experiments on the “defective” table were limited by the fact that the antecedent *A* and consequent *C* of the conditional did not have the third value, but de Finetti's 3 × 3 table, while encompassing the 2 × 2 de Finetti table, allows *A* and *C* to have the third value. In brief, we can find in the 3 × 3 de Finetti tables an answer to the question of what value does *if A then C* have when *A* or *C* have the third value (even though this was not his main objective). Of course, because the 3 × 3 table incorporates the 2 × 2 table, it keeps answering the question of what value does *if A then C* have when *A* is false: it is void. Note that *void* defined by a state of ignorance (as well as *irrelevant* expressed by participants in psychological experiments) is not a truth value homogeneous with *true* and *false*; rather, it is a meta-evaluation (for an analysis of this point, see Dubois and Prade, 2001; Dubois, 2008). It is in this sense that the 3 × 3 logic is superimposed on the 2 × 2 logic.

The conditional is so important that Baratgin et al. (2013) initially focused on it in their experimental study of three-valued tables. They observed that almost 60% of participants who gave responses in agreement with de Finetti's 2 × 2 table expanded it to produce de Finetti's full 3 × 3 conditional event table, when evaluating indicative conditionals and conditional bets. This is the first result supporting de Finetti's Level 1 system, but it is limited. Extending it to the other connectives of the system would demonstrate its descriptive adequacy, that is, *provide a semantic theory of the interpretation of natural language connectives under uncertainty*. This is the objective of the present paper. But before proceeding to the experiments, we should make some theoretical and methodological points. There exist many three-valued logical systems (for reviews, see Rescher, 1969; Haack, 1974; Gottwald, 2015). Some of them appeared before de Finetti, and many more have appeared in cognitive science since then. Psychologists of reasoning have so far done little to study whether any of these tables matches the judgments of ordinary people when they are in a state of ignorance about what is true and what is false (but see Elqayam, 2006, on “liar” paradoxes)^3^. Some of these systems propose a conditional table encompassing the 2 × 2 de Finetti table and so constitute possible alternative theories to de Finetti's Level 1 system, Fi. We give a short overview of these systems in the next section. See Appendix A (Supplementary Material) for a more detailed description, and Appendix B (Supplementary Material) for a presentation of the authors' individual reasons for developing their systems.

## Nine systems of three-valued tables

### An extension of 2 × 2 Bi-valued tables

In addition to de Finetti's (1936/1995) 3 × 3 table for the conditional event, there exist numerous other possibilities to build a 3 × 3 table to represent the indicative conditional of natural language, which we will call the *natural conditional*. Consider column 4 in Table 1 in which *C*|_?_*A* represents a general 3 × 3 conditional table for this natural conditional. Here *A* and *C* can be true (“T”), false (“F”), or judged to be neither. After lines 1, 3, 7, and 9 have been filled in with the values of the “defective” table, there remain five cells marked with “?.” The basic question is: what value should be in the place of each “?” to represent the natural conditional? There are 243 possible ways (3^5^), in theory, of completing this conditional table. The same question is also posed for the other connectives. Among the existing three-valued logics we have found only nine three-valued systems that extend the 2 × 2 de Finetti table for the conditional and that also propose 3 × 3 tables that extend standard two-valued logic for the conjunction and disjunction connectives. By “extending,” we mean 3 × 3 tables that have the same *true* or *false* values as their 2 × 2 counterpart in lines 1, 3, 7, and 9 mentioned above. Looking for such extensions is motivated by the experimental evidence that the classical 2 × 2 conjunction and disjunction truth tables are produced by a majority of people (Manktelow, 2012).

These nine systems of three-valued tables originate from the work of logicians, linguists, philosophers, and artificial intelligence researchers, who had different theoretical interests and approaches. As we will see in section Interpreting the Connectives and Appendix B (Supplementary Material), this is most evident in their interpretation of the third value. Some of these systems were not originally intended to represent an intuitive sense of uncertainty, which de Finetti aimed to capture (Baratgin and Politzer, 2016), but even so, they do have some *prima facie* interest for psychological modeling, simply because they extend the traditional 2 × 2 tables of two-valued logic to three-valued systems. Three-valued judgments have long been found in truth table studies of the conditional in psychological research, as we have described.

In summary, there are four basic connectives (negation, conditional, conjunction, disjunction). Three types of conditional (see Table 1, columns 5, 6, and 7) and four types of conjunction and disjunction (see Appendix A, Table A.2 in Supplementary Material) constitute the *differential* building blocks of the nine three-valued systems: as displayed in Appendix A, Table A.3 (Supplementary Material), each system is defined by using the involutive negation and by selecting one type of connective among the other three basic connectives^4^. A short reminder on the origins of three-valued logic is given in Appendix B (Supplementary material), followed by the origins of the nine “extended” systems [numbered (1)–(9)].

### Interpreting the connectives

The different truth tables for the connectives in Table 1 and Tables A.1, A.2, A.5 (Supplementary Material) may appear somewhat formal, and so we give a brief informal overview of how they differ from each other. We begin with the conditional *if A, then C*.

Recall that six systems, (1)–(6) in Appendix B (Supplementary Material), adopt the Fi conditional and so share the notion that a conditional with a false antecedent takes on the value ∅. Indeed, we have already seen through the betting schema that, whenever the antecedent A is not known to be true (∅ or F), the Fi conditional takes on the value ∅. In addition, a conditional sentence whose antecedent is true takes on the truth value of its consequent.

What distinguishes the Fi conditional from the other two conditionals, in columns 6 and 7 of Table 1, appears precisely for the value ∅ of the antecedent in lines 4 and 6. Two of the nine systems, (8) and (9) in Appendix B (Supplementary Material), use the Cooper conditional. For this conditional, with a truth-value gap ∅ (denoted by G for gap by Cooper) for the antecedent, the conditional takes on the value of the consequent, which is also the case when the antecedent is T. This captures the notion that the conditional has the same value with a ∅ antecedent as it has with a T antecedent. Only when the antecedent is F is the conditional ∅ whatever the value of the consequent. The Farrell conditional, (7) in Appendix B (Supplementary Material), differs in that it adopts a slightly more cautious evaluation: When the antecedent has a truth-value gap ∅ (denoted by I for ignorance by Farrell) and the consequent is T the conditional is not T but ∅ (Table 1, column 6). Note that both concur in holding the conditional to be F when the antecedent is ∅ and the consequent F. How this differs with Fi can be exemplified as follows. Suppose it is unknown whether *this chip is square*, while it is false that *this chip is black*. Then to evaluate *if this chip is square, then it is black*, some theorists (like Farrell and Cooper) may have the intuition that it is “false,” whereas others (like de Finetti) may have the intuition that the value is “void”^5^.

We can further examine the differences between systems by comparing the four types of conjunction and disjunction on which they are based that we have identified, viz., KLH, B, S, and M [defined in Appendix A (Supplementary Material)]. Most proposed systems (like Fi) in Appendix B (Supplementary Material) have truth-value gaps and consequently differ from three-valued systems proper in which the third value is homogeneous with the values T and F to which it can be compared using a relation of order. Most authors define an order between the truth-value gap and T and F. In de Finetti's framework, the truth-value gap is viewed as intermediate between F and T. Mura (in de Finetti, 1995/2008) gives a pragmatic justification with the bet schema: the payoff of a void bet is clearly intermediate between the payoff of a bet that is lost and a bet that is won (for more technical justification, see Hailperin, 1996; Milne, 1997, 2004; Blamey, 2001; Mura, 2016). It is exactly the order of KLH connectives. Conjunction obeys the following principle: the three values are formally put in an order denoted by *F* ≤ ∅ ≤ *T* (Dubois and Prade, 1994); then, whenever two sentences are connected, the value of the conjunction is the minimum of their values, that is, the conjunction gets the “weaker” value. Consider a context of chips of different shapes and colors. With the interpretation of ∅ as a truth-value gap resulting from ignorance, take a true sentence, for instance *the chip is square* (T), and suppose one is ignorant whether *the chip is black* (∅); then the conjunction *the chip is square and it is black* is evaluated as ∅ because min(T, ∅) = ∅. Suppose now *the chip is square* to be false; then the conjunction *the chip is square and it is black* is evaluated as F because min(F, ∅) = F. Similar considerations obtain for disjunction, *mutatis mutandis*. Here the value of the connection is defined by the maximum values of the disjuncts. If it is known to be true that *the chip is square* (T) and one is ignorant whether *the chip is black* (∅), then *the chip is square or it is black* will be evaluated as true because max(T, ∅) = T.

The various conjunctions obey the min order but they have their own formal order for the three values, which in fact differentiates them from each other. The same obtains for the disjunctions with the max order. We will not review them in detail, but will have a look at what the choice of an order intuitively means. Take Bochvar's (1938/1981) conjunction ∧_*B*_. Its order is ∅ < F < T. This means that whenever a sentence with the third value is connected (conjunctively) with another sentence whatever its value, the third value prevails and “contaminates” the conjunction. Similarly for disjunction ∨_*S*_, the order corresponds to F < T < ∅. For instance, with a third value interpreted as *of no interest*, the sentence *the chip is square* being true or false and *the chip is black* being of no interest, the sentence *the chip is square or it is black* will be evaluated as being of no interest in each case. The situation is opposite for the Sobocinsky connectives where the orders are F < T < ∅ for conjunction and ∅ < F < T for disjunction, resulting in connections that appear to be “immune” to the third value as the other values absorb it. With the previous example, the disjunction will be evaluated as T in the first case and F in the second one.

Finally, consider involutive negation, which all the systems share. T is negated by F and F by T like in two-valued logic. Negating the third truth value by itself captures the intuition that one cannot consider a sentence that is not ∅ as T any more than consider it as F, so that it remains ∅.

*Prima facie* all the nine systems, irrespective of their origins and motivations, provide candidates for three-valued tables relevant to the psychological modeling of people's comprehension of connectives under uncertainty. They accommodate and extend the 2 × 2 de Finetti table for the conditional, which is supported by earlier psychological research, as we have explained. Most of the systems above are directly relevant to psychologists, especially those motivated by linguistic considerations and the inappropriateness of the material conditional to represent people's interpretation of the natural language conditional (such as BFM, etc., defined in Appendix A (Supplementary Material). Clearly, an empirical investigation is necessary to decide which of these three-valued systems best fits ordinary people's judgments about natural language connectives. We present several experiments that aim to answer this question by examining people's truth tables for negation, conjunction, disjunction, the conditional, and the biconditional. More strongly, we ask whether the tables closest to people's judgments belong to one system in the literature. For all the reasons detailed in section The de Finettian Approach, and in view of the results we have already obtained for the conditional, we consider de Finetti's Level 1 system as the most serious contender. Recall that it is characterized by the Fi conditional and the KLH conjunction and disjunction.

## Experiments: the finettian and other three-valued systems

### Method

#### Participants

In Experiment 1 (*N* = 54) and Experiment 2 (*N* = 101), participants were French native speakers. They were students at the University Paris 8 who volunteered for the experiments. They already held a degree and were resuming their studies in a remote teaching program in the social sciences. They had no specific background in logic or probability theory. In Experiment 3, participants were 58 undergraduate Japanese native speaker students enrolled in a computer programming class at the Tokyo Denki University. All were naive to the purposes of the study. Experiments 1 and 2 were administered on a computer screen and Experiment 3 was presented in a booklet. An online informed consent was obtained from all participants. This study was carried out in accordance with the recommendations of the APA ethical principles and code of conduct and was approved by the ethics committee of Laboratoire Cognitions Humaine et Artificielle (*EA 4004–CHArt*), Université Paris 8, France.

#### Materials

**Figure 1:**
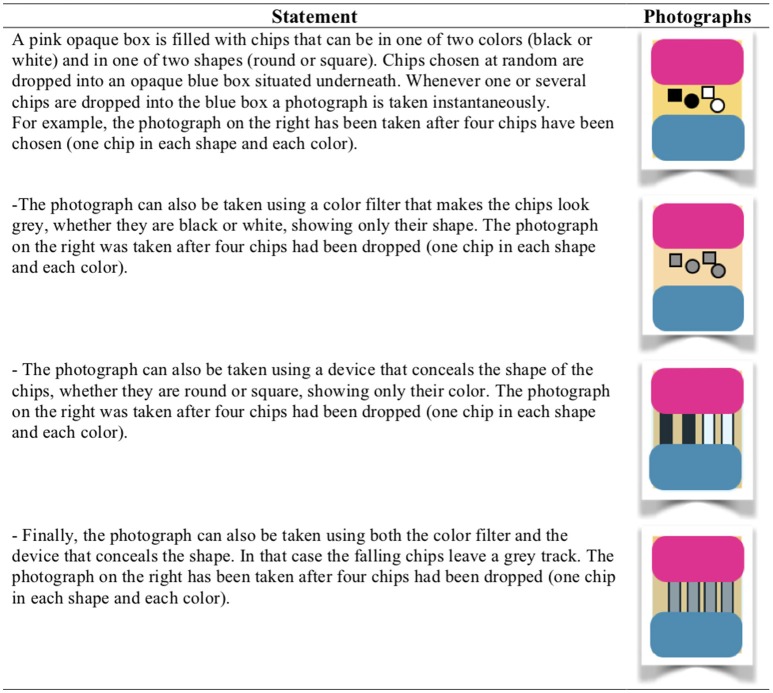
The presentation of the game in Experiments 1 and 2.

In Experiment 1 and 2 the same material was used. Participants were presented with sentences that referred to a chip that could be in one of two colors, black or white, and one of two shapes, square or round. The task was to judge whether the sentences were true, false, or neither. There were different conditions of visibility. In one condition, the chip was seen through a transparent window, making it clearly true whether the chip was square, or round, and similarly making it clearly true whether the chip was black or white. In another condition of visibility, the chip was seen through a device that made it visually impossible to know whether the chip was square or round. And in a third condition of visibility, the chip was seen through a filter making it visually impossible to know whether the chip was black or white. And finally the chip could be seen through both the device and the filter, making both the shape and the color impossible to identify. This technique allowed us to fill up the nine cells of a three-valued truth table with the participants' responses (see Figure 1).

**Figure 2:**
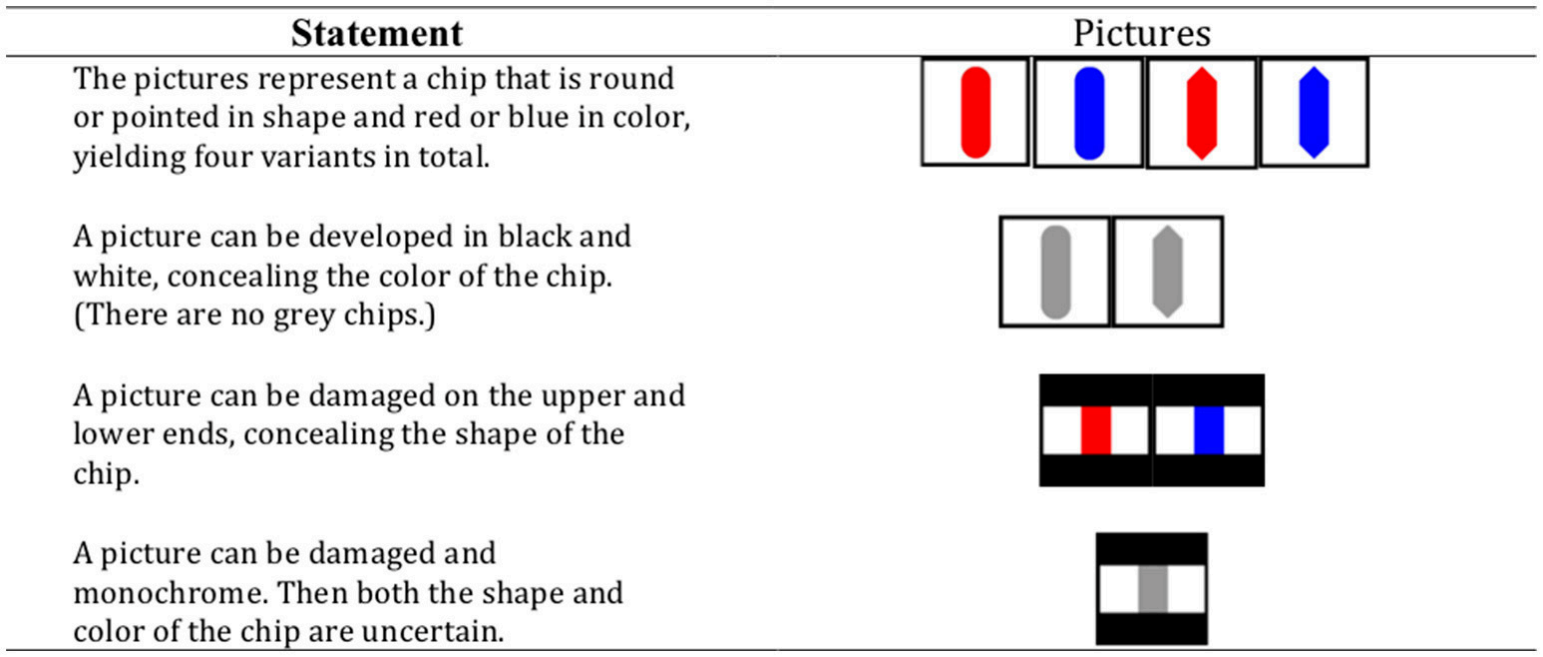
The presentation of the game in Experiment 3.

In the third experiment (Japanese participants), an isomorphic material with pictures of round or pointed chips that could be blue or red was used (Figure 2).

#### Design and procedure

**Figure 3:**
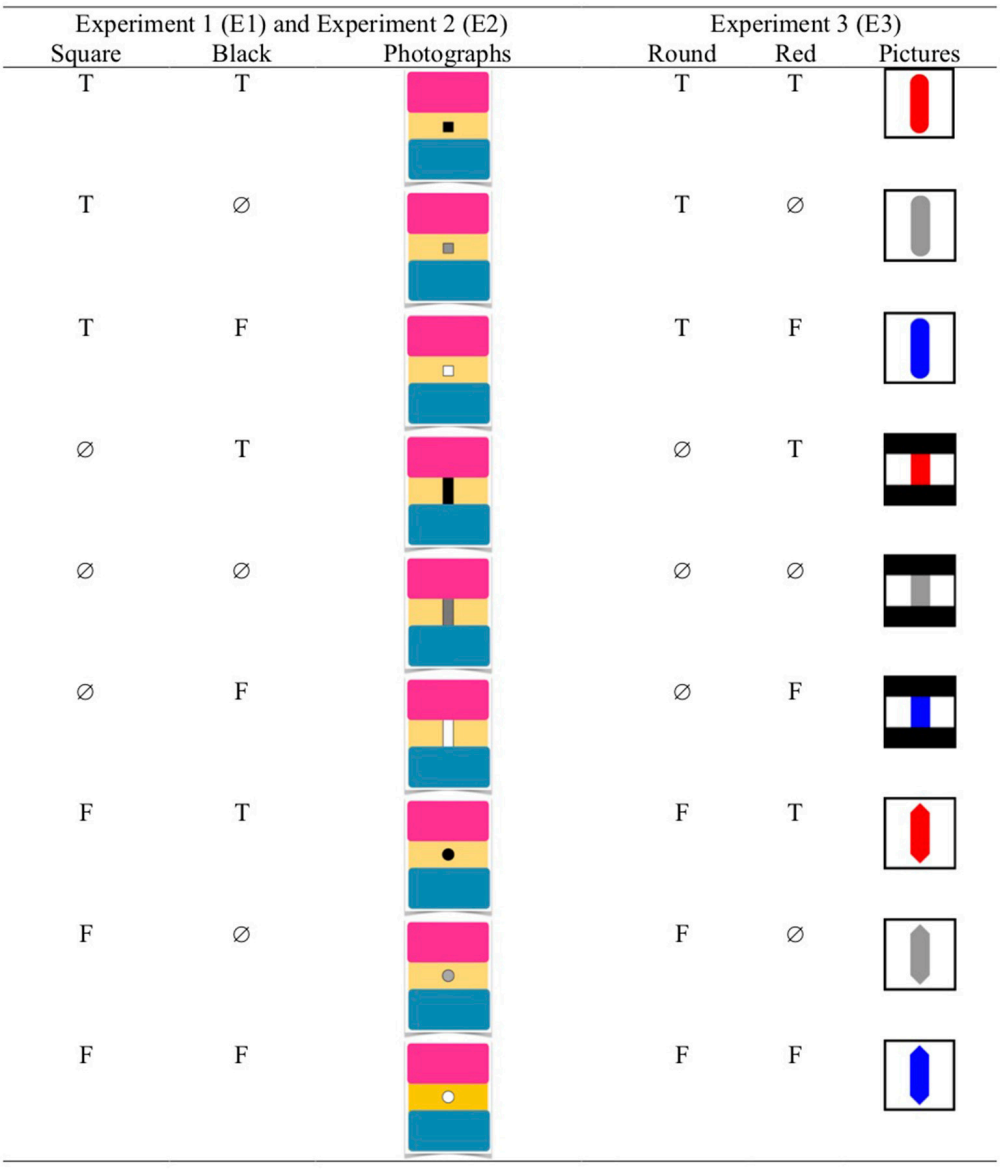
The nine possible combinations of photographs (Experiments 1 and 2) or pictures (Experiment 3) corresponding to the nine cells of the three-valued truth table.

In the three experiments, participants were required to judge the truth value of the sentence under consideration for the nine combinations corresponding to the nine cells of the truth table (see Figure 3).

**Figure 4:**
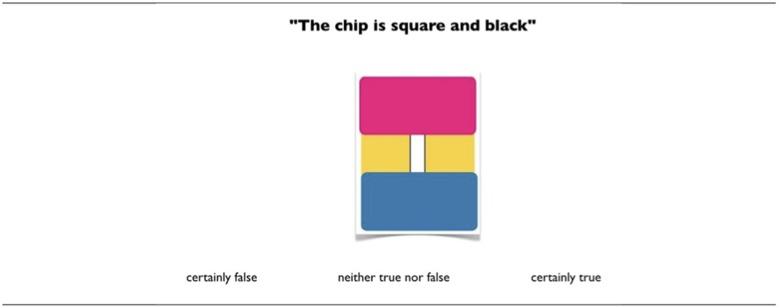
Example of a trial (one logical combination) for conjunction. The first conjunct has the third value ∅ and the second conjunct is false.

For the three experiments the combinations were presented in a random order and each one was accompanied by three response options: *certainly true, certainly false, neither true nor false*. The participants were required to select one option (see an example in Figure 4 for the conjunction).

The choice of the adverb “certainly” reflects the Finettian notion that when an event is known to have occurred or not to have occurred, this is known with certainty, and so the truth or falsity of the proposition that expresses it is certain. Besides, this should avoid possible common fuzzy interpretations of “true” and “false” such as “very likely to be true/false.” This wording has already been used for the same purpose in the context of research on the framing effect (Mandel, 2014).

The choice of the wording *neither true nor false* for the third option was made for several reasons. First it should be as close as possible to de Finetti's conception and formulation of the third value. This third value (or *void*, as de Finetti often called it) is the evaluation made by an individual who is not in a position to know whether an event is true or false. Commenting on the tri-event, de Finetti explicitly states that the third value is to be regarded as neither true nor false: ”Whenever the *condition B* is satisfied, then *A|B* is either true or false (1 or 0). But unless the condition *B* is satisfied, one can neither say that the event *A|B* is true, nor that the event *A|B* is false. It is *void* or *null* in the sense that the premise under which it is considered either true or false no longer holds. In my opinion, these three cases should be treated as distinct“ (de Finetti, 1995/2008, p. 170).

Second, it should capture participants' natural evaluations, that is, with as little suggestion as possible. In principle, the third option could be “true or false” (or some equivalent expression such as “It could be true or it could be false.” This option is correct (and trivial) from a logician's objective point of view. But it does not readily accommodate subjective judgments, in particular those generated by three-valued systems (see the various and subtly different interpretations of the third value in section Interpreting the Connectives). By parity of argument, it might be objected that “neither true nor false” cannot accommodate the choice of “true or false” because it is incompatible with it. This is correct, but pragmatically rejecting the assertions that the sentence is true and that it is false gives rise to the assertion that it is neither. More precisely, participants who do not find an assertable option are led to interpret the third option as a means to express just this (and to disregard the logical triviality in case it had come to their mind). The judgment that neither “true” nor “false” are adequate options induces the judgment that “neither true nor false” is adequate, which turns the third option into a meta-option equivalent to “other” that cannot be put on the same level as “true,” “false,” and “true or false.”

Third, the format should be common to all the connectives. For the conditional in particular, it should be possible for participants to express a judgment such as “void” or “irrelevant” without any suggestion, which the “neither” option satisfies. Note that the first constraint above is exemplified with the conditional which theoretically returns the value *void* in case its antecedent is not known to be true, but the other connectives also have logical cases of voidness for which the option “neither” is appropriate for the same reasons. In brief, the aim of the third option is to capture the judgment that neither the first option nor the second is adequate, in the spirit of de Finetti, without influencing the participants, while being applicable to the various connectives, and the formulation adopted does just that.

In Experiment 1, each participant was asked to judge the truth value of a negated sentence (e.g., *the chip is not square* when the shape of the chip presented could be square or round or indeterminate, and the color black, white or indeterminate), hence nine presentations (or “trials”). Participants were randomly allocated to one of the two statements, *the chip is not square* and *the chip is not black*.

In Experiment 2, each participant received four sentences: first the simple affirmation, *The chip is a square* to familiarize them with the task. This was followed by a conjunction, *The chip is square and black*; then there were two sentences presented in a counter-balanced order: a disjunction disambiguated by “or both” written in parentheses, *The chip is square or black (or both)*^6^, and a conditional, *If the chip is square, then it is black*. The conditional will not be detailed here (for the results, see Baratgin et al., 2013).

In Experiment 3 (Japanese sample), each participant received four sentences, in this order: the simple affirmation, *The chip is red*, the conjunction, *The chip is round and red*, the conditional, *If the chip is round, then it is red*, and the biconditional, *If the chip is round, then it is red, and if it is red, then it is round*. The original sentences in French and Japanese can be found in Appendix C (Supplementary Material).

### Results

#### Method of analysis

The tables produced by participants will be analyzed in two stages. The first stage casts the results in terms of the traditional two-valued classification. That is, we restrict the analysis of the answers to the four “old” cells of the traditional table that correspond to the four cases where the antecedent and the consequent are either true or false. This allows the identification of a 4-cell truth table for each connective and each participant (and a 2-cell truth table in the case of negation). In this way, we take up the classic 2 × 2 tables before extending them into new 3 × 3 tables.

In the second stage of the analysis, we further characterize the tables by considering all nine cells (and all three cells for negation). Then the observed three-valued tables are compared with the relevant three-valued formal tables of the nine systems.

#### The first stage analysis

Table 2 displays for each connective the frequency distribution of the interpretations (the tables produced) in percent. To answer the research question, we were basically interested in the identification of the modal response, that is, we were looking for a dominant interpretation belonging to the same system across connectives. In each of the first three columns there is one modal response >70% (close or equal to 100% in the first three columns), that is, a clearly dominant response appears. However, in the last two columns (conditional and biconditional) the modal response is not so high. To identify this modal response as a reliable dominant interpretation, a 95% confidence interval for proportions (based on z values) was calculated (rounded to the closest unit) for all percentages >10%. Confidence intervals will also be given for the second stage analysis.

**Table 2:** First stage analysis.

**Connective** **Tables produced**	**Negation** **E1**	**Conjunction** **E2 and E3**	**Disjunction** **E2**	**Conditional** **E3**	**Biconditional** **E3**
Negation ¬*A* (*or* ¬*C*)	100 [94; 100]				
Conjunction A∧C		98 [93; 100] (Experiment 2) 98 [90; 100] (Experiment 3)		22.4 [14; 35]	20.7 [12; 33]
Disjunction A v C			73.3 [64; 81]		
Conditional “defective” C|_″*d*″_*A*				37.9 [27; 51]	1.7
Material conditional *A*⊃*C*				3.4	
Material biconditional *A*⇔*C*				15.5 [8; 27]	25.9 [16; 39]
Biconditional *C*||*A*				15.5 [8; 27]	50.0 [38; 63]
Other		2 (Experiment 2)2 (Experiment 3)	26.7 [19; 36]	5.1	1.7

*Frequency distribution of the tables produced (in percent) for the five connectives considering only two truth values for A and C. In brackets: 95% confidence intervals. E1, Experiment 1, N = 54; E2, Experiment 2, N = 101; E3, Experiment 3, N = 58*.

For negation (experiment 1), all participants answered in agreement with the two-valued truth table of negation.

For the conjunctive statement, 98% of the participants in Experiment 2 as well as in Experiment 3 respected the conjunction table.

For the disjunctive statement 73.3% respected the disjunction table. These rates correspond to the traditional rate of response presented in the literature. In particular, the review made by Evans et al. (1993) for disjunction shows that the true-false combinations are evaluated as false between 10 and 28% of the time, indicating a conjunctive interpretation. Similarly, virtually all of the 27 participants who did not respect the standard truth table answered *false* to the true-false combinations, either on one occasion (20) or on both (6). This means that these participants had difficulty processing disjunction and had a tendency to construe it as a conjunction in line with the classic results, and that their error was not due to having trouble with the uncertain cases or with the response format, that is, with the three-valued system. Finally, there was no case of exclusive interpretation, indicating that the disambiguation by “or both” was effective.

For the conditional statement, the two main tables produced by Japanese participants of Experiment 3 correspond to the usual “defective” conditional (37.9%) and conjunction tables (22.4%). These frequencies are comparable to Baratgin et al. (2013) French data. The only notable difference is that the frequency of the biconditional table which was virtually null now reaches 15.5%.

For the biconditional statement, the dominant interpretation is the 2 × 2 de Finetti table (50%), followed by the material biconditional table (25.9%) and the conjunction table (20.7%).

#### The second stage analysis

We consider all nine cells of the observed truth tables. Each participant's table is classified by considering the formal table to which it is the *closest*. Our criterion of “closeness” or “distance” is as follows. A participant's table is taken to be a perfect instance of a formal table X when it is identical to X. A participant's table is a “close” instance of X when it differs from X just by one cell, and *from any other formal table by more than* one *cell*. If a participant's table differs equally (by one cell) from two (or more) formal tables, it is still “close” to, but classified as *ambiguous* between, these tables (these are equally likely). Finally, if a participant's table differs by two or more cells from all formal tables, then it is classified as “indeterminate”: it differs too much to make a reliable identification.

First of all, for the simple affirmation, *the chip is square*, all participants answered correctly, that is, *certainly true* when the chip was square, *certainly false* when it was round, and *neither true nor false* when its shape was blurred. This is evidence that the square, blurred, and round shapes were visually well distinguished, allowing participants to recognize the three logical possibilities, and in particular, the representation of uncertainty by the blurred image. Importantly, there was a perfect one-to-one correspondence between the blurred image and the *neither* answer, which validates this formulation.

For the negation, 48 participants (89%) fully conformed to the involutive negation ¬_*i*_ (in which the third value maps onto itself) on all nine trials, and six participants (11%) answered in agreement with this table on eight trials (meaning that they were closer to the involutive negation than to any other type of negation). Two of these six participants clearly made a well-known slip triggered by double negation (Wason, 1959), answering F instead of T to a round chip when the sentence was *The chip is not square*. The other four participants negated the ∅ chip by answering F (three cases) or the T chip by answering ∅ (just for one case). The answers provided by these four participants are thus closest to an involutive negation than to left and right negations. In brief, we find evidence of only the involutive negation.

Before considering conjunction and disjunction, note that the numbers for these two connectives are smaller than they are in the first stage. This is because the three-valued tables for conjunction and disjunction (like for negation) are built as expansions of the corresponding classic two-valued tables which serve as filters, so that only participants who have produced the latter can be considered in the second stage. For instance, we have mentioned earlier that 98% of the 101 participants in Experiment 2 (i.e., 99 participants) produced a conjunction table in the first stage analysis. Consequently, the second stage analysis for conjunction and the related percentages are based on those 99 participants. The only case where N is notably diminished is disjunction in Experiment 2 (from 101 to 74, as mentioned in section The First Stage Analysis). The results for conjunction and disjunction are detailed in Tables 3, 4 in which we will examine the sum column.

**Table 3:** Second stage analysis. Conjunction.

**Conjunction tables produced**	(0)^*^		(1)^**^		**Sum (0)**+**(1)**	
	**E2**	**E3**	**E2**	**E3**	**E2**	**E3**
*KLH* (C∧_*K*_*A*)	76.7	96	6.1	0	82.8 [74; 89]	96 [88; 99]
*McCarthy* (C∧_*M*_*A*)	13.1	4	0	0	13.1 [8; 21]	4
*Other*	4.1				4.1	0

Frequency of tables produced (in percent) considering three truth values.

* (0), 0 difference (all nine cells coincide);

** *(1), one difference (8 cells coincide). In brackets: 95% confidence intervals. E2, Experiment 2, N = 99; E3, Experiment 3, N = 57. The Table reads as follows: in Experiment 2, 76.7% of the 99 participants produced the exact KLH table, and 6.1% produced it with one difference, so that 82.8% produced the KLH table with at most one difference, with a 95% confidence interval of [74; 89], etc*.

**Table 4:** Second stage analysis. Disjunction.

**Disjunction tables produced**	(0)^*^	(1)^**^	**Sum (0) + (1)**
*KLH* (C∨_*K*_A)	52.7	5.4	58.1 [48; 69]
*Sobocinsky* (C∨_*S*_A)	8.1	6.8	14.9 [9; 25]
Ambiguous (1 difference with ∨_*K*_ and with ∨_*S*_)		16.2	16.2 [12; 29]
Other			10.8 [8; 22]

Frequency of tables produced (in percent) considering three truth values. Experiment 2, N = 74.

* (0), 0 difference (all nine cells coincide);

** *(1), one difference (8 cells coincide). In brackets: 95% confidence intervals*.

For conjunction (Table 3), it is apparent that a large majority of the observations coincide with the *KLH* connective ∧_*K*_ defined in Appendix A, Table A.2 (Supplementary Material) (82.8% in Experiment 2 and 96% in Experiment 3). The remaining interpretations correspond to the McCarthy ∧_*M*_ conjunction (13.1 and 4%, respectively).

For disjunction (Table 4), the absolute majority of the observations (58.1%) coincide with the *KLH* connective ∨_*K*_ defined in Appendix A, Table A.2 (Supplementary Material). The remaining interpretations coincide with the Sobocinsky disjunction ∨_*S*_ (14.9%) and to tables that we call “ambiguous” because they differ equally (by one cell) from both disjunctions ∨_*K*_ and ∨_*S*_.

For the conditional (Table 5), almost all the participants' interpretations coincide with a table that belongs to the Fi system. We find notably that all of the 22 participants whose first stage table was identified as de Finetti's 2 × 2 “defective” table expanded this table into de Finetti's conditional event (3 × 3) table. In other words, the conditional probability to produce the three-valued conditional event table knowing that the two-valued table is the “defective” table equals one. Also of interest is the fact that most participants (84.6%) who have a conjunctive interpretation of the conditional in the first stage expand this table into a conjunction table (the KLH table) that is in the Fi system. These results confirm the observations of Baratgin et al. (2013) with French participants. Similarly, most participants giving a biconditional interpretation produce the Fi biconditional. Interestingly, even for the material biconditional interpretation, most participants produce the associated Finettian table.

**Table 5:** Second stage analysis. Conditional and biconditional.

	**Conditional**			**Biconditional**		
**Tables produced**	(0)^*^	(1)^**^	**Sum (0) + (1)**	**(0)**	**(1)**	**Sum (0) + (1)**
Conditional			*N = 22* (37.9%)			*N = 1* (1.7%)
*de Finetti (C*|_*Fi*_*A*)	95.5	4.5	100 [85; 100]	100		100
Conjunction			*N = 13* (22.4%)			*N = 12* (20.7%)
*KLH* (*A*∧_*K*_*C*)	53.8	30.8	84.6 [58; 96]	75	8.3	83.3 [55; 95]
*Other*	15.4		15.4 [4; 42]	16.7		16.7 [5; 45]
Material conditional			*N = 2* (3.5%)			
*Kleene (A*⊃_*K*_*C*)	50		50			
*Other*	50		50			
Material biconditional			*N = 9* (15.5%)			*N = 15* (25.9%)
*Kleene (A*⇔_*K*_*C*)	77.8	11.1	88.9 [56; 98]	66.7	20	86.7 [62; 96]
*Other*			11.1 [2; 44]	13.3		13.3 [4; 38]
Biconditional			*N =* 9 (15.5%)			*N = 29* (50%)
*de Finetti (C*||_*Fi*_*A*)	77.8		77.8 [45; 94]	93.1	6.9	100 [88; 100]
*Other*			22.2 [6, 55]			
Other			*N =* 3 (5.2%)			*N = 1* (1.7%)

Frequency of tables produced (in percent) considering three truth values. Experiment 3, N = 58.

* (0), 0 difference (all nine cells coincide);

** *(1), one difference (8 cells coincide). In brackets: 95% confidence intervals. The Table reads as follows: for the conditional, 22 participants (out of 58 = 37.9%) produced a conditional table that was identical to de Finetti's table and no other conditional table was observed; still for the conditional, 13 participants (out of 58 = 22.4%) produced a conjunction table; 11 of these (84.6%) produced a KLH table; and 2 (15.4%) produced a different conjunction table, etc*.

For the biconditional sentence (Table 5), the observations are identical: almost all the participants' interpretations coincide with a table that belongs to the Fi system. In particular, the dominant biconditional interpretation is always the Finettian one, that is, 100% of 3 × 3 de Finetti biconditional table. Similarly, most participants (86.7%) with a material biconditional interpretation choose the expanded Kleene material biconditional [defined in Appendix A, Table A.5 (Supplementary Material)] and also most of those (83.3%) with a conjunctive interpretation produce the associated Finettian table [the KLH conjunction ∧_*K*_ defined in Appendix A, Table A2 (Supplementary Material)]. All this suggests a remarkable consistency within a unique logical system, namely the Fi system.

We can summarize these results as follows. The overwhelmingly dominant table for *A and C* is the KLH conjunction ∧_*K*_ and the dominant table for *A or C* is the KLH disjunction ∨_*K*_, both of which are features of the Finettian system. Whatever the interpretation for *if A then C* (conditional, conjunction, biconditional, material biconditional), it is the corresponding Finettian table that is overwhelmingly the dominant choice. This obtains also for *if A then C and if C then A*, whatever its interpretation (conjunction, biconditional, material biconditional). In addition, the involutive negation is always observed.

## Discussion

### De finetti's level 1 system as the best approximation

The hypothesis that de Finetti's Level 1 system is adequate to model the psychological three-valued truth tables for natural language connectives is clearly supported by the results in the following two respects. One, its constitutive connectives: involutive negation ¬_*i*_, the KLH conjunction ∧_*K*_, the KLH disjunction ∨_*K*_, the Fi conditional *C*|_*Fi*_*A* and the Fi biconditional *C*||_*Fi*_*A*, have been found to be the dominant interpretations. This was the case for the two languages studied, French and Japanese, which offers a remarkable cross-linguistic support to the Finettian theory on Level 1, given the remoteness of the two linguistic families. Two, even when the conditional and biconditional sentences are not construed as a conditional or a biconditional, respectively, the truth table that is produced still belongs most generally to the Fi system. However, it can be objected to the first point that the other two logical systems that are built on the same connectives, namely McDermott, and Reichenbach could, *eo ipso*, be regarded as possible candidates. Is there a way to decide between the three systems? We have seen earlier that the latter two differ from de Finetti in that they have additional connectives.

Consider first Reichenbach's system, (2) in Appendix B (Supplementary Material). It has additional connectives (two more negations), and two material conditionals and two material biconditionals [see Tables A.1 and A.4 (Supplementary Material)]. We made no observation of a form of negation other than the involutive one, nor did we find any trace of the two forms of material conditional or biconditional. We can conclude that Reichenbach's three-valued logic is inadequate in that it predicts several truth tables, that is, interpretations of the negation, conditional, and biconditional, that our participants never had. This is not too surprising given that the objective of his logic is to account for a problem that belongs to the epistemology of quantum mechanics. Even though there is striking overlap between his system and the three-valued table of the Finettian conditional, the additional connectives needed for his purpose are irrelevant for psychological modeling. To take but one example of the lack of plausibility of the system from a psycholinguistic point of view, the cyclical negation of A requires a triple application of the operator to get back to A: *A* = ~~~ *A*; and the complete negation holds only as: $\bar{A}=\bar{\bar{\bar{A}}}$, whereas double negation does apply to diametrical (involutive) negation: *A* = ¬_*i*_¬_*i*_*A* (see Table A.1).

McDermott's system, (4) in Appendix B (Supplementary Material), also has additional connectives: one conjunction (∧_*S*_) and one disjunction (∨_*S*_). For disjunction, we did find some trace of ∨_*S*_ (15%, against 58% for ∨_*K*_), but for conjunction we did not find any trace of ∧_*S*_. This does not support the system. However, before eliminating it, we must envisage that there may be special conditions or circumstances under which the second set of connectives is used, which our material may have failed to meet. McDermott (1996) contented himself to remark, based on intuition, that *and*, and *or* are ambiguous in natural language, hence his definition of two different connectives in each case. But to exemplify the ambiguity he did not use simple sentences made of two atomic components, such as *A and B*, or *A or B*. Instead, he used complex sentences, one component of which was always a conditional (such as *A and if B then C*, or *A or if B then C)*. Obviously, if this is required for the supplementary connectives to apply, the double connective claim cannot be refuted by our experimental results, which are based on at most two atomic sentences. McDermott's theory is not specified enough in its current state and the question remains open for further research. But it should be noted that if the claim becomes experimentally supported, it would come as an extension of the Finettian system proper. It is remarkable that McDermott's approach has much in common with de Finetti's, in particular in the assessment of truth values using the betting method, and crucially in the definition of the natural conditional. Finally, the conditions that trigger the additional connectives interpretation could have a pragmatic explanation, keeping the Finettian system semantically unaltered. For all these reasons, the objections to the first point above seem hard to maintain; in addition they leave the second point unaffected. This is why we can confidently conclude that our results designate the Finettian system as the best approximation to the participants' three-valued truth tables obtained from judgments of truth and falsity of atomic sentences describing uncertain characteristics.

### The significance of the results: logic and the study of human reasoning

Our results constitute a step toward giving an integrated answer to three related questions. One, is there a dominant interpretation of the basic connectives with sentences that have a truth-value gap? Two, do these interpretations constitute a consistent system? Three, is there a way to solve the half-a-century-old problem of the “defective” truth table of the conditional?

#### The existence of a dominant interpretation

We have obtained an affirmative answer to the first question. For each connective (negation, conjunction, disjunction, conditional, and biconditional), participants' interpretations were distributed over a limited number of table varieties among numerous possible tables, and for each connective, there was a clear dominant interpretation, namely, involutive negation, the KLH conjunction ∧_*K*_, the KLH disjunction ∨_*K*_, the Fi conditional *C*|_*Fi*_*A* and the Fi biconditional *C*||_*Fi*_*A*, respectively. For negation there was a single interpretation (involutive). For conjunction the modal interpretation (∧_*K*_), collapsed over two experiments, was close to 90%. For disjunction the modal interpretation (∨_*K*_) was chosen 58% of the time (among participants who had a 2 × 2 disjunctive interpretation) while the next most frequent interpretation was seldom chosen (16%). For the conditional and the biconditional, one interpretation (*C*|_*Fi*_*A* and *C*||_*Fi*_*A*, respectively) was chosen 100% of the time (among participants who had the corresponding 2 × 2 interpretation). In brief, given people's two-valued interpretation, there is always one way to extend this interpretation to a three-valued table that musters an absolute majority, and (except for disjunction) there is near unanimity for this interpretation.

#### The existence of a system

We have obtained an affirmative answer to the second question too: not only is there a predominant interpretation for each connective, but this interpretation always belongs to the same system. It could have been the case that while the dominant table for one connective belongs to one system, the dominant table for another connective belongs to another system. But this is not what we have observed: for each connective, out of all the possible tables, it is the one that belongs to the Finettian system that dominates. And there is more: even when the two-valued table has deviating interpretations, which occurs for the conditional and the biconditional, the table is almost always completed into the corresponding Finettian table. See for instance how in Experiment 3 the two-valued conjunctive interpretation of the biconditional made by 12 participants (20.7%, Table 2, first stage analysis) leads ten of them (83.3%) to the corresponding conjunctive Finettian (∧_*K*_) three-valued interpretation shown in Table 5 (second stage analysis). All this means that the present results are more than an extension to the other connectives of the results obtained for the conditional by Baratgin et al. (2013). Rather, what we have established here is *the existence, in people's judgments under uncertainty, of mutually consistent interpretations of the standard connectives organized in* one *system, namely de Finetti's Level 1 system*.

#### Interpreting the “defective” table

Finally, we have obtained confirmation of a positive answer to the third question, “Can the puzzle of the defective table be solved?” Three-valued truth tables generalize two-valued tables. They collapse into two-valued tables when the component sentences are certain. In such a case, for the conditional, the third value ∅ left in the body of the 2 × 2 table constitutes the “defective” table and the explanation of its origin. Note that there is no conflict between the two-valued and three-valued tables. The latter incorporate the former in the same way that rational numbers include integers.

### The significance of the results: interpreting the third value

We recalled some important findings in the introduction. For several decades psychologists have known that people judge that *if A then C* is true when *A* holds and *C* holds, false when *A* holds and *C* does not, and neither true nor false when *A* does not hold. For the last decade, there has been growing psychological evidence that people tend to judge that the probability of the indicative conditional, *P(if A then C)*, is the conditional probability of *C* given *A, P(C|A)*. There is also evidence supporting the claim that people tend to be coherent in explicit deduction under uncertainty. More recently, psychologists have shown that there is a close relation between indicative conditionals and conditional bets (Oberauer and Wilhelm, 2003; Politzer et al., 2010; Baratgin et al., 2013, 2014; Nakamura et al., 2018). There is an urgent need to integrate these experimental findings. The integration has been held back because psychologists did not raise the general question of which three-valued tables correspond most closely to people's judgments under uncertainty.

In the present paper, we have raised the question and proposed an answer based on de Finetti's Level 1 system, offering a model of the interpretation of natural language connectives under uncertainty. Obviously, we should keep in mind the limitations of our study due to the size of the samples and more importantly to the fact that the sentences in the experiments referred to specific materials. No overall investigation of the foundations of de Finetti's system, at Level 1, had yet been carried out. In view of the psychological relevance and plausibility of de Finetti's subjective approach to probability, and of the successful application of his concepts and ideas recalled above, it would have been deeply puzzling if the interpretation of connectives had been found to be at variance with his system. But on the contrary, our results showing that people conform to de Finetti's Level 1 system add much support to the project of developing the psychology of reasoning on a de Finettian basis, within the Bayesian account of ordinary reasoning that we are pursuing.

Our results should lead to more research. Important questions concerning the interpretation of the third value still await investigation. We have seen that the various systems of three-valued logic have different uses and objectives in defining a third value (even though most systems studied interpret it as a truth-value gap, see Appendix B (Supplementary Material). However, there appears to be an underlying common notion, that of doubt about truth and falsity due to uncertainty.

Even though we have identified the most frequent interpretation of each connective, namely the one that belongs to the Finettian system, it must be kept in mind that this result relies on a few experiments that operationalized uncertainty as ignorance about which of two values a visually defined variable had. Visual uncertainty about the identification of two shapes or two colors was hypothesized to coincide with de Finetti's concept of a “void” judgment. We certainly acknowledge that there is a need for additional experiments using other languages and, more importantly, that vary the source of uncertainty. There are perhaps other types of visual uncertainty, e.g., arising from soritical series (see Douven et al., 2018), and we should move beyond the visual modality, e.g., to sound or haptic modalities, and then beyond the sensory modalities, e.g., to logical or semantic uncertainty (as can be found in the paradoxes of self-reference; see Elqayam, 2006). The choices are unlimited, for uncertainty is everywhere in natural language, a point de Finetti himself would have emphasized. If the results of such experiments are consistent with the present observations, then there will be stronger support for the general conclusion that the Fi system is the best semantic theory of the interpretation of natural language connectives under uncertainty.

In contrast, it is possible to operationalize a much more common concept of uncertainty. Considering that ignorance reflects a lack of information that could be dispelled as information increases, still using the same material participants could be provided with frequency distributions about the proportions of round, square, black, and white chips, that is, manipulating the base rates. With this additional knowledge, one is invited to move from radical uncertainty to a gradable notion of uncertainty in which the individuals' degrees of belief vary between 0 and 1. In this modified situation there is no more total ignorance and the individual shifts from Level 1 to Level 2. But in doing so, one would be losing the state of total ignorance whose investigation is the objective of the present work, and as noted earlier (section The de Finettian Approach) the psychological research on reasoning under uncertainty (and indeed a large amount of research on judgment and decision making under uncertainty) has essentially been carried out on Level 2.

We have considered the basic connectives, but it might be interesting for future research to study more complex sentences than the basic ones. Conditionals can be embedded like in left embedding *If they were outside (O), then if it rained (R) they got wet (W)* or in right embedding *If the cup broke (B) if dropped (D), then it was fragile (F)* (see Gibbard, 1981; Douven and Verbrugge, 2013; Douven, 2016). In the Finettian framework of Level 1, these sentences can be written as (*W*|_*Fi*_*R*)|_*Fi*_O and *F*|_*Fi*_(*B*|_*Fi*_*D*), respectively and they collapse into a single form, *W*|_*Fi*_(*R*∧_*K*_*O*) and *F*|_*Fi*_(*B*∧_*K*_*D*), respectively (de Finetti, 1974, p. 328). Their truth tables can be established, allowing a further test of the theory on its Level 1 (van Wijnbergen-Huitink et al., 2015). Recently the Finettian treatment of embedded conditionals on level 2 has attracted the attention of theorists (Gilio and Sanfilippo, 2014; Douven, 2017; Sanfilippo et al., 2018) with results that reflect the difference of perspective between the two levels.

One final remark also for future research: in the current study we have compared various systems by eliciting judgments of truth value for connected sentences. Given that each system has a consequence relation, another way to test the systems against each other could be to study the elementary inferences that reasoners are willing to make.

## Author contributions

JB and GP: design of the study and data analysis. JB and TT: data collection. GP and JB: draft of the manuscript. JB, GP, and DO: conceptual elaboration. DO and TT: critical revision of the manuscript.

### Conflict of interest statement

The authors declare that the research was conducted in the absence of any commercial or financial relationships that could be construed as a potential conflict of interest.

## Footnotes

^1^Several philosophers have proposed an identical 2 × 2 “defective” table in their analysis of “if” in ordinary language, with different interpretations of the third value. According to Quine (1950) a conditional affirmation with a false antecedent is as if it had never been made. O'Connor (1951) defines a table with a third undetermined value. Dummet (1958/1959) presents a similar table where the third value corresponds to neither true nor false. Kneale and Kneale (1962, p. 135–136) suggest a “defective” table (in the sense of *defective truth function*) where the value I (denoted by ”–“) characterizes a truth-value gap.

^2^There is much more in the betting scheme: de Finetti (1937/1964) proposed it at Level 2 as a procedure to operationally evaluate P(C|A), from which it follows that the conditional event can be represented as a three-valued random quantity taking on values 1, 0, P(C|A) (Gilio, 1990). Note that de Finetti (1962, 1964/1972, 1974) proposed also the penalty criterion (based on the Brier score) as a procedure to operationally evaluate P(C|A) (see for a recent analysis Gilio and Sanfilippo, 2011).

^3^Stenning and Van Lambalgen (2008) used Kleene's (1938) three-valued logic in the framework of logic programming, but they did not study participants' truth tables.

^4^With the exception of the R system, the material conditional and the material biconditional are not defining features because they can be derived compositionally from the basic connectives using the formulas *A* ⊃ *C* =_*df*_ ¬ *A* ∨ *C*, and *A* ⇔ *C* =_*df*_ (*A* ⊃ *C*) ∧ (*C* ⊃ *A*).

^5^One may balk at this notion because conditionals typically have uncertain antecedents and nevertheless they often convey a high degree of belief (or disbelief) rather than ignorance. One need not know whether it is true that *this man will fall from the 20th floor* to hold it to be false that *if this man falls from the 20th floor he will survive*. In this apparent counter example, which is on Level 2, the common knowledge suggests a degree of belief. In contrast, with our abstract and arbitrary material on Level 1 individuals have no expectations about the truth value of the conditional.

^6^In doing so, we followed psychologist's traditional way of disambiguating “or” in the study of reasoning.
