# Supplementary material for: The Psychology of Uncertainty and Three-Valued Truth Tables
Source: Front Psychol. 2018 Sep 4;9:1479. doi: 10.3389/fpsyg.2018.01479 (PMC6131665; doi:10.3389/fpsyg.2018.01479)
Supplement: Supplementary file 1 [file Presentation_1.pdf]

Supplementary Material

Appendix A. The constitution of the nine three-valued systems

Here we describe the constitution of the nine three-valued systems (defined in Section 3.1), which means essentially defining their connectives and how this differentiates them from each other.

The negation. All nine systems have the *involutive* negation (denoted by $\neg_i$). It is defined as follows: when A is true, $\neg_i$A is false; when A is false, $\neg_i$A is true; and when A takes on the third value ∅, so does $\neg_i$A. There exist other definitions of negation (see Table A.1).

Table A.1 *The different types of negation and their truth tables in three-valued logic (columns 1 to 5) together with other unary connectives (columns 6 to 10).*

	1	2	3	4	5	6	7	8	9	10
A	$\neg_i$A	~A	Ā	$\neg_l$A	$\neg_r$A	T(A)	H(A)	Δ(A)	∂(A)	F(A)
T	F	∅	∅	F	F	T	T	∅	T	F
∅	∅	F	T	F	T	F	F	∅	∅	∅
F	T	T	T	T	T	F	T	F	∅	F

Note. 1. $\neg_i$A: the involutive negation. 2. ~A: Reichenbach's "cyclic negation". 3. Ā: Reichenbach's "complete negation". 4. $\neg_l$A : the "left" negation. 5. $\neg_r$A: the "right" negation. 6. T(A): de Finetti's unary Thesis connective. 7. H(A): de Finetti's Hypothesis connective. 8. Δ(A): Hailperin's unary connective "don't Care". 9. ∂(A): Beaver's unary operator. 10. F(A): Cantwell's unary connective "it is false that A".

The conditional. Six of the nine systems, (1) - (6) in Appendix B, extend de Finetti's 2x2 table for the conditional by using a 3x3 conditional table identical to de Finetti's ($C|_{Fi}A$, Table 1, column 5). System (1) is Fi itself, but these six systems differ from each other by having different conjunctions or disjunctions (see below). The last three systems, (7) - (9) in Appendix B, extend de Finetti's 2x2 table for the conditional by using a 3x3 conditional table that differs from de Finetti's. We call one of them, in (7) of Appendix B, the *Farrell* conditional table ($C|_{Fa}A$, Table 1, column 6). The remaining two systems, (8) and (9) in Appendix B, have what we will term the *Cooper* conditional table in common ($C|_{C}A$, Table 1, column 7). They differ from each other by having different conjunctions and disjunctions.

Conjunction and disjunction. The nine systems select their conjunctive and disjunctive connectives among four types that were defined earlier by various researchers working on three-valued logic. They are the following (their truth tables are given in Table A.2):
(i) *Kleene-Łukasiewicz-Heyting* (henceforth KLH), denoted by $\wedge_K$ and $\vee_K$
(ii) *Bochvar* (internal), denoted by $\wedge_B$ and $\vee_B$
(iii) *Sobocińsky*, denoted by $\wedge_S$ and $\vee_S$
(iv) *McCarthy*, denoted by $\wedge_M$ and $\vee_M$.

Table A.2. *Three-valued truth tables for conjunction* (∧) *and disjunction* (∨).

A	C	$A \wedge_K C$	$A \wedge_B C$	$A \wedge_S C$	$A \wedge_M C$	$A \vee_K C$	$A \vee_B C$	$A \vee_S C$	$A \vee_M C$
T	T	T	T	T	T	T	T	T	T
T	∅	∅	∅	T	∅	T	∅	T	T
T	F	F	F	F	F	T	T	T	T
∅	T	∅	∅	T	∅	T	∅	T	∅
∅	∅	∅	∅	∅	∅	∅	∅	∅	∅
∅	F	F	∅	F	∅	∅	∅	F	∅
F	T	F	F	F	F	T	T	T	T
F	∅	F	∅	F	F	∅	∅	F	∅
F	F	F	F	F	F	F	F	F	F

Note. $\wedge_K$, $\vee_K$: Kleene-Łukasiewicz-Heyting; $\wedge_B$, $\vee_B$: Bochvar; $\wedge_S$, $\vee_S$: Sobociński; $\wedge_M$, $\vee_M$: McCarthy.

The constitution of the nine systems in terms of the basic types of connectives (conditional, conjunction, disjunction) is shown in Table A3. We denote the third value by ∅ for all the systems to avoid increasing the number of symbols.

Table A.3. *The constitution of the nine systems (left column) in terms of the types of conditional, conjunction, and disjunction connectives.*

	Conditional			Conjunction				Disjunction			
	Fi	Fa	C	K	B	S	M	K	B	S	M
(1) Fi	+			+				+			
(2) R	+			+				+			
(3) BG	+				+					+	
(4) BFM	+					+				+	
(5) McD	+			+		+		+		+	
(6) MBV	+						+				+
(7) FGNW		+		+				+			
(8) CBSAC			+			+				+	
(9) CaGO			+	+				+			

Note. The types of connectives:
Fi: de Finetti; Fa: Farrell; C: Cooper; K: Kleene-Łukasiewicz-Heyting; B: Bochvar; S: Sobocińsky; M: McCarthy.
The nine systems (see Appendix B):

1. Fi: de Finetti (1936)
2. R: Reichenbach (1944)
3. BG: Bruno & Gilio (1985)
4. BFM: Belnap (1970); Farrell (1986); Mura (2016)
5. McD: McDermott (1996)
6. MBV: Muskens, Van Benthem, & Visser (1996)
7. FGNW: Farrell (1979); Goodman, Nguyen, & Walker (1991)
8. CBSAC: Cooper (1966); Belnap (1973); Calabrese (1987)
9. CaGO: Cantwell (2008); Grandy & Osherson (2014)

Material conditional and material biconditional. Once the systems (denoted generically by X) have been defined and differentiated by their conditional, conjunction, and disjunction connectives, they can be enriched by their internal *material conditional* connective $A \supset_{\mathrm{X}} \mathrm{C}$. This connective is an extension of the four logical cases of the 2x2 material conditional ⊃; the way the five empty cells (that is, the other five logical cases) are filled up will again yield different truth tables (see Table A.4). Similarly a *material biconditional* connective $\Leftrightarrow_{\mathrm{X}}$ can be defined (based on the conjunction of $A \supset_{\mathrm{X}} \mathrm{C}$ and its converse $\mathrm{C} \supset_{\mathrm{X}} A$ like in two-valued logic, see Table A.4).

Table A.4. *Truth tables for the material conditional (⊃) and the material biconditional (⇔).*

A	C	$A \supset_K C$	$A \supset_B C$	$A \supset_S C$	$A \supset_M C$	$A \supset_L C$	$A \supset_{Be} C$	$A \Leftrightarrow_K C$	$A \Leftrightarrow_S C$	$A \Leftrightarrow_L C$	$A \Leftrightarrow_R C$
T	T	T	T	T	T	T	T	T	T	T	T
T	∅	∅	∅	F	∅	∅	F	∅	F	∅	F
T	F	F	F	F	F	F	F	F	F	F	F
∅	T	T	∅	T	∅	T	T	∅	F	∅	F
∅	∅	∅	∅	∅	∅	T	T	∅	∅	T	T
∅	F	∅	∅	F	∅	∅	T	∅	F	∅	F
F	T	T	T	T	T	T	T	F	F	F	F
F	∅	T	∅	T	T	T	T	∅	F	∅	F
F	F	T	T	T	T	T	T	T	T	T	T

Note. For the material conditional: $\supset_K$: Kleene; $\supset_B$: Bochvar; $\supset_S$: Sobociński; $\supset_M$: McCarthy ($\supset_{\mathrm{L}}$, $\supset_{Be}$ for the R system)
For the material biconditional $\Leftrightarrow_K$: Kleene/Bochvar/McCarthy (identical); $\Leftrightarrow_S$: Sobociński ($\Leftrightarrow_{\mathrm{L}}$, $\Leftrightarrow_{\mathrm{R}}$ for the R system).

Biconditional. With two exceptions, there is no definition in the nine systems of a biconditional connective based on the conditional (that would extend the "defective" biconditional $\mathrm{C}||_{\text{"d"}}\mathrm{A}$). However, from a psychological point of view, if people naturally interpret the natural language conditional following the conditional table, they must also interpret the natural biconditional as the conjunction of two conditionals. It is then important to introduce the biconditional in the systems in which it is lacking. This supplements the systems without logical alteration, because the biconditional is defined using two connectives already defined, the conjunction and the conditional following the formula: $\mathrm{C}||_{X}\mathrm{A} =_{\mathrm{df}} \mathrm{C}|_{\mathrm{X}}\mathrm{A} \wedge_{\mathrm{X}} \mathrm{A}|_{\mathrm{X}}\mathrm{C}$. As far as the Fi system is concerned, Gilio, Over, Pfeifer and Sanfilippo (2017) showed that (using our notations) the following equalities obtain: $\mathrm{C}||_{\mathrm{Fi}}\mathrm{A} = \mathrm{C}|_{\mathrm{Fi}}\mathrm{A} \wedge_{\mathrm{K}} \mathrm{A}|_{\mathrm{Fi}}\mathrm{C} = (\mathrm{A} \wedge_{\mathrm{K}} \mathrm{C})|_{\mathbf{Fi}}(\mathrm{A} \vee_{\mathrm{K}} \mathrm{C})$. The different biconditional connectives for the nine systems are presented in Table A.5.

Table A.5. *The biconditional truth tables of nine three-valued logical systems.*

A	C	1<br>$C\|\|_{"d"}A$	A	C	2, 3, 4<br>$C\|\|_{Fi}A$<br>$C\|\|_{BFM}A$<br>$C\|\|_{McD}A$	5<br>$C\|\|_{BG}A$ †	6<br>$C\|\|_{MBV}A$ †	7, 8<br>$C\|\|_{FGNW}A$<br>$C\|\|_{FCaGo}A$	9<br>$C\|\|_{CBSAC}A$
T	T	T	T	T	T	T	T	T	T
			T	∅	∅	∅	∅	∅	T
T	F	F	T	F	F	∅	∅	F	F
			∅	T	∅	∅	∅	∅	T
			∅	∅	∅	∅	∅	∅	∅
			∅	F	∅	∅	∅	F	F
F	T	F	F	T	F	∅	F	F	F
			F	∅	∅	∅	∅	F	F
F	F	∅	F	F	∅	∅	∅	∅	∅

Note.

1. $C||_{"d"}A$ defective (two-valued)
2. $C||_{Fi}A =_{df} (C|_{Fi}A \wedge_K A|_{Fi}C)$ Fi and R systems
3. $C||_{BFM}A =_{df} (C|_{Fi}A \wedge_S A|_{Fi}C)$ BFM system
4. $C||_{McD}A =_{df} (C|_{Fi}A \wedge_K A|_{Fi}C)$ McD system
5. $C||_{BG}A =_{df} (C|_{Fi}A \wedge_B A|_{Fi}C)$ BG system
6. $C||_{MBV}A =_{df} (C|_{Fi}A \wedge_M A|_{Fi}C)$ MBV system
7. $C||_{FGNW}A =_{df} (C|_{Fa}A \wedge_K A|_{Fa}C)$ FGNW system
8. $C||_{FCaGo}A =_{df} (C|_{C}A \wedge_K A|_{C}C)$ CaGO system
9. $C||_{CBSAC}A =_{df} (C|_{C}A \wedge_S A|_{C}C)$ CBSAC system

For the systems 2, 3, 4, 7 and 8 (denoted by X) the following relation obtains:
$C||_{X}A =_{df} (C|_{X}A \wedge_X A|_{X}C) = (A \wedge_X C)|_{X}(A \vee_X C)$

† The tables for $C||_{BG}A$ and $C||_{MBV}A$ do not extend the 2x2 defective biconditional table $C||_{"d"}A$.

Appendix B. The origin of three-valued logics and the nine "extended" systems

In a range of natural circumstances, people cannot readily classify sentences as true or false. Aristotle made this point in his discussion of future-contingent sentences such as (S1) *there will be a naval battle tomorrow.* He argued that declaring this sentence definitely true or false now can only be done at the risk of fatalism. Another circumstance pointed out by Frege (1892/1952) concerns sentences that contain non-referring singular terms, such as (S2) *Ulysses was set ashore at Ithaca while sound asleep.* Declaring the sentence true or false requires that Ulysses have a reference, failing which the sentence is neither true nor false. However, these insights did not lead to the elaboration of logical systems. Rescher (1969) identifies MacColl (1837-1909), C. S. Pierce (1839-1914) and N.A. Vasiliev (1880-1940) as the first authors to have proposed systems of propositional logic where propositions can have more than two values. Reflecting on Aristotle's case of future-contingent sentences, Łukasiewicz (1920/1967) proposed a third truth value, and he did formulate a full three-valued system that challenged standard two-valued logic. It notably rejects the logical principles of the excluded middle and non-contradiction. The three values are *true* (denoted by 1), *false* (denoted by 0), and a third value often denoted by ½, which represents what is possible. In 1922, Łukasiewicz extended his three-valued logic to a many-valued logic, in which a degree of possibility is given a number between 0 and 1. Other circumstances than contingency or lack of reference can result in the attribution of a third truth value. In 1938, Bochvar's investigation of the semantic paradoxes resulted in a three-valued logic, in which the third value indicates that the sentence is undefined or meaningless. For instance, a version

of the Liar paradox (S3) *this sentence is a lie* can be neither true nor false, since assuming that it is true implies that it is a lie and assuming that it is false implies that it is true. In 1938, Kleene's work on recursive functions in mathematical logic resulted in the elaboration of a three-valued logic in which the third value refers to what is *non-decidable*. Non-decidability can be thought of as a radical type of uncertainty in mathematics. There is a distant relation between Kleene's work and ours, but our notion of uncertainty does not come from a lack of mathematic information, but rather from limited visual information. More important for our purpose is the investigation of conditionals in logic and linguistics. The question of the interpretation of a conditional sentence whose antecedent is false led de Finetti (1935, 1936) to the definition of the *conditional event*, which is *void* in such a case, that is, it cannot be classified as true or false. (It can be declared true or false only in case its antecedent is true). As can be seen, different authors pursued quite different objectives, and accordingly the conception of the third value varied widely. One dividing line separates the third value considered on a non-epistemic par with truth and falsity, as in Łukasiewicz's logic, and the third value as an epistemic absence of truth or falsity, as in de Finetti's logic. In de Finetti, the third value, "void", is superimposed on bivalent logic and is given an epistemic interpretation, which we operationalized in our experiment as uncertainty caused by a lack of visual information. Ultimately in de Finetti, the third value becomes the full range of subjective probabilities. For introductions to many-valued logic, see Haack (1974) and Rescher (1969), and for discussions of the interpretation of the third value, see Cobreros, Egré, Ripley and van Rooij (2014), Gottwald (2015), and Rescher (1962).

The nine "extended" systems (1) to (9)

Six systems, (1) - (6), out of the nine formally adopt de Finetti's conditional event table

The 3x3 de Finetti conditional event table ($\mathrm{C}|_{\mathrm{Fi}}\mathrm{A}$, Table 1, column 5) has been constructed by several authors. Most of these authors failed to attribute the table to de Finetti; they actually rediscovered the table, giving various interpretations of the third value depending on their research field. Here we consider six three-valued systems that are comprehensive, starting with de Finetti's original Level 1 system Fi (with five additional versions of it numbered (a) to (e)), followed by five systems coming from formal logic or philosophical logic.

(1) The Fi system

As laid out by de Finetti, the Fi system includes the involutive negation $\neg_i$, the conjunction $\wedge_K$ and the disjunction $\vee_K$ as well as the material conditional $\supset_K$. (There are two additional connectives that allow to fall back on a two-valued system: a Thesis connective T(A) which means "A is true", and a Hypothesis connective H(A) which means "A is not null", $\mathrm{X} = \mathrm{C}|_{\mathrm{Fi}}\mathrm{A}$; $\mathrm{T(X)} = \mathrm{C} \wedge \mathrm{A}$ and $\mathrm{X} = \mathrm{T(X)}|_{\mathrm{Fi}}\mathrm{H(X)}$ (see Table A.1, columns 4 and 5). Fi has been used by several authors (Baioletti & Capotorti, 1994, 1996; Coletti & Scozzafava, 2002; Milne, 1997; Mura, 2009; Rothschild, 2014). Some authors have defined an expansion of the Fi system that maintains tautologies of bi-valued logic (such as "$\neg_{\mathrm{i}}\mathrm{A} \vee_{\mathrm{K}} \mathrm{A}$", see Mura for a discussion). In this approach Baioletti & Capotorti (1994, 1996) equipped Fi with two additional negations called "left" (denoted by $\neg_l$) which yields F when ∅ is negated and "right" (denoted by $\neg_r$A) which yields T when ∅ is negated[1] (see Table A.1, columns 4 and 5). We present below the different authors who have concurred to propose a system formally identical to Fi system, but motivated by different objectives and offering various interpretations of the third value. The following five systems are logicians' creations.

[1] The left negation corresponds to Bochvar's "external negation" and to Heyting's negation, and the right negation corresponds to "F-split" negation presented in Rescher (1969) and to "Improved Negation" introduced in Bourne (2004). Left and right negations correspond respectively to "radical" and "classical" negation of Seuren (1988).

(a) Hailperin (1996, 2011) developed a probability logic called "sentential suppositional logic" in which $C|_{Fi}A$ has a suppositional interpretation: "C, supposing A" or "Supposing A, then C". The third truth value denoted by "u" represents a value "either true or false, it is undetermined, unknown or of no interest which" (Hailperin 2011, p. 29). Hailperin introduces a connective Δ (see Table A.1, column 6) which means "don't care" and can be used to define $C|_{Fi}A$ as follows: $C|_{Fi}A =_{df} \Delta(\neg_i A) \vee_K (C \wedge_K A) =_{df} \mathrm{Max}\{\mathrm{Min}\{A, C\}, \mathrm{Min}\{1 - A, u\}\}$. This system is analogous to Milne's (2004) system where $C|_{Fi}$ is defined in the same way: $C|_{Fi}A =_{df} (N \wedge_K \neg_i A) \vee_K (C \wedge_K A))$ with N as a "sentential constant" which always takes the value ∅.

(b) Blamey (2001) introduced a "simple partial logic", where the third value "✶" is interpreted as a truth-value gap, an undefined value "neither true nor false" that cannot be compared to the two truth-values true and false. In this system $C|_{Fi}A$ is called the "transplication". Blamey uses in addition an "interjunction" connective (denoted by "xx"). The conditional can be defined in relation to the interjunction by:

$C|_{Fi}A =_{df} (A \wedge_K C) \times\times (\supset_K C)$.

(c) Ellis (1973) discusses a logic of subjective probability identical to Fi where the three truth values are based on a notion of empirical truth. T means "is accepted as empirically true", F means "is accepted as empirically false" and "X" means "is accepted as empirically undecided". $C|_{Fi}A$ is again introduced by the bet analogy.

(d) Rescher (1962, 1969), reflecting on students' difficulty to learn and understand the material conditional when the antecedent is false, discusses a quasi-truth functional system where the third truth value (denoted by "(T, F)") is defined as an "undetermined" value which can be either T or F depending on the circumstances, denoted by (T, F).

(e) Beaver (1992, 1997; Beaver & Kramer, 2001) develops in linguistics an approach of presupposition by formalizing a three-valued logic with a third value (denoted by "N" for neither); this applies in particular when a statement that carries a presupposition is produced in a context where the presupposition is not satisfied, the statement's truth-value must be undefined. In Beaver's logic, $C|_{Fi}A$ is used as an "elementary presupposition operator" that can be read as "C with presupposition A". In case A is not true, then $C|_{Fi}A$ takes on the value N. $C|_{Fi}A$ is defined with a "unary operator" denoted by ∂ (see Table A.1, column 7) by:

$C|_{Fi}A =_{df} (\partial(A) \wedge_K C) \wedge_K (\partial(A) \wedge_K \partial(\neg_i A))$.

(2) The R system

The second system is called R for Reichenbach's quantum logic (Reichenbach, 1944, 1952/1953). Reichenbach introduces a "quasi-implication" identical to $C|_{Fi}A$ which is an extension of a 2x2 "quasi-implication" identical to de Finetti's conditional event. A standing for "a measurement of the entity is made" and C for "the measured value is C" the quasi-implication can be verified or falsified only if A is true, and is *indeterminate* or *meaningless* (denoted by "I") if A is false. There is an obvious and remarkable formal analogy with de Finetti's conditional event that can be true or false only if A is true, and is void if A is not true (see the appendix of de Finetti, 1974). The system includes the involutive negation (called "diametrical negation") and two other negations ("cyclical" ~A, and "complete" $\bar{A}$, see their definition in Table A.1). It includes the KLH conjunction $\wedge_K$ and disjunction $\vee_K$. In addition, it contains two material conditionals: $\supset_L$ and $\supset_{Be}$, and two material biconditionals: $\Leftrightarrow_L$ and $\Leftrightarrow_R$ (borrowed from systems that we are not considering here because they belong to the class of systems that do not accommodate a conditional extending the conditional event).

(3) The BFM system

The BFM system was presented independently as the logic of assertion by Belnap (1970), as a logic of presupposition by Farrell (1986) and as a generalization of de Finetti's logic to avoid counterintuitive results of de Finetti's system has been proposed by Mura

(2016). In Belnap (1970), $C|_{Fi}A$ corresponds to a conditional assertion (the assertion of C on the condition A in a possible world w). The third value represents the case where the conditional is "unassertive" (undefined). If A is true at world w, what the assertion of C on the condition A is what C asserts at w. If A is false or non assertive at w, then the assertion of C on the condition A is non-assertive at w. In Farrell (1986), the third value (denoted by "I") means the "to be ignored". Recently Mura (2016) proposed a similar system based on $C|_{Fi}A$ that allows (with a new definition of logical consequence) to maintain tautologies (such as "A|A"). In the BFM system, the conjunction and the disjunction correspond to $\wedge_S$ and $\vee_S$, respectively.

(4) The McD system

This system was introduced in philosophical logic by McDermott (1996) to account for the truth conditions of "if" sentences. Independently of de Finetti's writings, McDermott proposes $C|_{Fi}A$ with a similar bet interpretation where the third value (denoted by "X") corresponds to a truth-value gap ("no truth value" when the bet is void). This three-valued logic has two conjunctions, $\wedge_K$ and $\wedge_S$, and two disjunctions, $\vee_K$ and $\vee_S$.

(5) The BG system

This system was developed by the mathematicians Bruno and Gilio (1985). It shares de Finetti's bet interpretation of the conditional. The difference with Fi consists in the disjunction and conjunction tables that correspond to $\vee_S$ and $\wedge_B$, respectively.

(6) The MBV system

MBV comes from the logic of presupposition literature (Muskens, Van Benthem, & Visser, 1996) where the third value represents an "undefined" value, and $C|_{Fi}A$ is defined as Blamey's transplication. The MBV system includes $\wedge_M$ as conjunction and $\vee_M$ as disjunction.

One system, (7), out of the nine formally adopts the Farrell conditional table

(7) The FGNW system

The Farrell conditional table is shown in Table 1 (column 6, $C|_{Fa}A$). Only one system, out of the nine that we are covering, uses this table. It has $\wedge_K$ and $\vee_K$ for conjunction and disjunction.

(a) This system was proposed first by Farrell (1979). His goal was to define an implication free of paradoxes (contrary to the material conditional). For Farrell, this third value "I" represents "inappropriate to assign truth value".

(b) An identical system (Chrząstowski-Wachtel, Tyszkiewicz, Hoffmann & Ramer, 2001) has been formulated independently by Goodman and colleagues in AI in the so-called GNW algebraic approach (Goodman, Nguyen, & Walker, 1991), where $C|_{Fa}A$ corresponds to a coset that can also be represented by an interval $[A \wedge_K C; \neg A \ \vee_K C]$. The third value "u" represents here the value "undefined".

Two systems, (8) - (9), out of the nine formally adopt the Cooper conditional table

The last two of the nine systems fall under the Cooper conditional table (Table 1, column 7, $C|_C A$).

(8) The CaGO system

The CaGO system includes $\wedge_K$ for the conjunction and $\vee_K$ for the disjunction. It is proposed in logic by several authors.

(a) First by Cantwell (2008, 2009) who interprets the third value as a "truth-value gap". He introduces $C|_C A$ as the natural connective that allows to define the involutive negation (called "inner negation", see Table A.1) interpreted as a form of "conditional" negation ($\neg_i A$ is read "*A* is false *if it has a truth value*"). Cantwell (2008) introduces three unary connectives, two of which correspond exactly to the de Finetti Thesis connective T(A) (which means for him "it is true that A"), and the Hypothesis connective H(A) (denoted by TV(A) which means "it is true

or false that A"). The third connective (denoted by F(A), see Table A.1) means "it is false that A". Cantwell shows that A is equivalent to $T(A)|_{C}TV(A)$ and $\neg A$ to F(A).
(b) The same system was proposed recently by Grandy and Osherson (2014) in their Sentential Logic where the third value (denoted by “U”) is considered as a "truth value gap.”

(9) The CBSAC system

The CBSAC includes the conjunction $\wedge_S$ and the disjunction $\vee_S$. It has been independently proposed by three authors motivated by different objectives.
(a) The linguist W. S. Cooper is the first to propose this system as a propositional logic of ordinary discourse (Cooper, 1968). The third value denoted by “G” represents a “truth value gap.”
(b) Belnap (1973), as a continuation of Belnap (1970) logic of assumption (see also Dunn, 1975), identified $C|_{C}A$ with a conditional assertion of C on the condition A in a possible world w. The third truth value still represents the case where the conditional is “unassertive” (undefined). Belnap (1973) proposes that the assertion of C makes an assertion iff A is not false (A is either true or non-assertive).
(c) Independently, in AI, Calabrese (1987, 2002) adopts an algebraic approach and defines an identical system where the third truth-value denoted by “U” is “the 'inapplicable’ or ‘undefined’ conditional”. In the AI literature, this system is called SAC for Schay, Adams and Calabrese. Adams and Schay are associated with this system because both authors independently introduced the connectives “quasi-conjunction” and “quasi-disjunction”, which are actually the Sobocińsky connectives. However these authors have never (to our knowledge) given explicitly the Cooper conditional table (with the nine cells) or an iterated rule of the conditional that produces the Cooper conditional table. Note also that Schay (1968) proposes two additional connectives for conjunction and disjunction that are the Bochvar (internal) connectives.

Note that we denote the third value by ∅ for all the systems to avoid increasing the number of symbols.

Appendix C. The original sentences in French (Experiments 1 and 2) and Japanese (Experiment 3)

Affirmation

E:	The	chip	is	square		
F:	Le	jeton	est	un carré		
	art	noun	verb	art+noun		
J:	Shashin-ni			utsutta	chippu-ha	akai.
	in the picture		photographed		chip	red

Negation

E:	The	chip	is	not	square
F:	Le	jeton	n'est pas		carré
	art	noun	neg+verb		adj

Conjunction

E:	The	chip	is	round and		red
F:	Le	jeton	est	rond	et	rouge

art noun verb adj conj adj

J:	Shashin-ni	utsutta	chippu-ha	marumatte ite	katsu,	aka-iro dearu.
	in the picture	photographed	chip	round	and	red

Disjunction

E:	The	chip	is	round	or	red	or	both
F:	Le	jeton	est	rond	ou	rouge	ou	les deux
	article	noun	verb	adj	conj	adj	conj	adv (art+numeral)

Conditional

E: If the chip is round then it is red

J:	Moshimo	shasin-ni	utsutta	chippu-ga		marumatte iru	naraba,
	if	in the picture	photographed	chip	is	round	then
	sore-ha	aka-iro dearu.					
	it is	red					

Biconditional

E: If the chip is round then it is red, and if the chip is red then it is round

J:	Moshimo	shasin-ni	utsutta	chippu-ga		marumatte iru	naraba,
	if	in the picture	photographed	chip	is	round	then
	sore-ha	aka-iro	deari, katsu				
	it is	red,	and				
	moshimo	shasin-ni	utsutta	chippu-ga		aka-iro	naraba,
	if	in the picture	photographed	chip	is	red	then
	sore-ha	marumatte iru.					
	it is	round.					
